# Objective Outcomes in Lateral Osteotomy Through Anterior-to-Psoas for Severe Adult Degenerative Spine Deformity Correction

**DOI:** 10.7759/cureus.18277

**Published:** 2021-09-25

**Authors:** Hasan S Ahmad, Andrew I Yang, Gregory W Basil, Michael Y Wang, Jang W Yoon

**Affiliations:** 1 Neurosurgery, Perelman School of Medicine, University of Pennsylvania, Philadelphia, USA; 2 Neurosurgery, University of Miami Miller School of Medicine, Miami, USA

**Keywords:** lateral osteotomy, lateral lumbar fusion, anterior-to-psoas, adult spinal deformity, digital health, smartphone, objective outcomes, spine surgery

## Abstract

Multilevel lateral interbody fusion is an acceptable surgical technique in patients with severe degenerative adult spinal deformity (ASD). The current standard-of-care in spine surgery includes the use of patient reported outcome measures (PROMs) to assess post-operative improvement. Objective activity data during the peri-operative period may provide supplementary information for patients recovering from ASD surgery. In this report, we use smartphone-based activity data as an objective outcome measure for a patient who underwent a two-stage operation for ASD corrective surgery: lateral osteotomy and lumbar interbody fusion with posterior column release.

An 82-year-old male presented with intractable back pain secondary to severe thoracolumbar scoliotic deformity (Lenke 5BN). Pre-operative images demonstrated the presence of bridging osteophytes over the left lateral aspect of L2-5 disc spaces and over the apex of the lumbar curvature, with significant neuroforaminal stenosis.

Surgical correction was completed in two stages: (1) left-sided lateral osteotomy using anterior-to-psoas approach (ATP) in a right lateral decubitus position, and (2) multilevel Ponte osteotomies and instrumented fusion from T10-pelvis. Post-operative radiography showed correction to scoliotic deformity and sagittal misalignment. The patient had developed seroma and wound dehiscence, which was evacuated on post-operative day 11. At 14-month follow-up, the patient reported significant improvement in pain symptoms, corroborated by patient reported outcome measures.

To further quantify and assess patient recovery, smartphone-based patient activity data was collected and analyzed to serve as a proxy for the patient’s functional improvement. The patient’s walking steps-per-day was compared pre- and post-operatively. The patient’s pre-operative baseline was 223 steps/day; the patient’s activity during immediate post-operative recovery dropped to 179 steps/day; the patient returned to baseline activity levels approximately 3 months after surgery, reaching an average of 216 steps/day.

In conclusion, we found that lateral osteotomy through an ATP approach is a powerful tool to restore normal spine alignment and can be successfully performed using anatomic landmarks. Additionally, smartphone-based mobility data can assess pre-operative activity level and allow for remote patient monitoring beyond routine follow-up schedule.

## Introduction

Adult spinal deformity (ASD) consists of a spectrum of pathologies commonly affecting the thoracolumbar spine, most frequently degenerative scoliosis [1]. Progression of spinal deformity can cause severe low back pain, leg pain, claudication, and sagittal imbalance. Altogether, these symptoms lead to reduced mobility, reduced ability to complete activities of daily living, and decreased overall quality of life.

Many options exist for surgical treatment of ASD; this choice can be informed by the specific class of scoliosis as determined by the Lenke classification [2], or by scoring systems that prognosticate patients and algorithmically select a surgical approach based on demographic factors such as age, BMI, bone quality, and degree of coronal and sagittal deformity [3,4]. Ultimately, however, the decision to undergo ASD corrective surgery is a deeply personal decision. This shared decision-making between patient and spine surgeon is critical for a successful outcome.

One potential operative approach for the correction of thoracolumbar degenerative scoliosis is through the following two-stage procedure: the first stage is a multilevel lateral approach to the apex of the lumbar curve for the correction of coronal deformity and the second stage includes additional posterior column osteotomies, to achieve further correction in both sagittal and coronal planes, and pedicle screw and rod insertion. Combined lateral and posterior approach for the correction of degenerative thoracolumbar scoliosis has several advantages over the posterior approach alone. First, it allows for significant coronal (11.7°) and sagittal deformity (2.9° per level) correction [5,6]. Second, it allows for arthrodesis in both anterior and posterior columns, which may lead to a more robust fusion construct and a lower rate of pseudoarthrosis, which can be as high as 35% [7]. Third, the surgeon can determine how much posterior column work needs to be performed based on the standing x-rays before the second stage posterior operation. Lastly, by breaking up the operation into two stages, the length of the operation may be reduced, lessening the burden on the patient as well as on the surgical team.

After surgical intervention, post-operative outcomes in spine surgery are often assessed using patient-reported outcome measures (PROMs) such as Oswestry disability index (ODI), EuroQOL-5D (EQ-5D), short-form health survey (SF-36), and PROM information systems (PROMIS) [8]. These PROMs assess a patient’s perspective on their disability and quality of life through the administration of static questionnaires (1) to understand the level of pre-operative baseline of disability, (2) to measure the impact of intervention based on pre-operative to post-operative score improvement, and (3) to conduct population-based analysis across multiple sites.

Despite their widespread use, the subjective and discrete nature of PROMs significantly impedes their efficacy in truly assessing functional spine surgery recovery and outcome [9]. Other groups have reported the use of stand-alone accelerometers to measure steps taken per day by patients pre- and post-operatively as a proxy for overall health and mobility in patients undergoing lumbar surgeries [10,11]. Our group has previously demonstrated that it is possible to use smartphone-based accelerometer data to objectively assess patients undergoing lumbar spine laminectomy and fusion [12]. This form of outcomes assessment utilizes a continuous and objectively calculated measure, and thus overcomes the main shortcomings of PROMs. In this study, we propose a novel method to objectively assess a patient undergoing a two-stage ASD corrective surgery.

## Case presentation

Patient information

**Figure 1:**
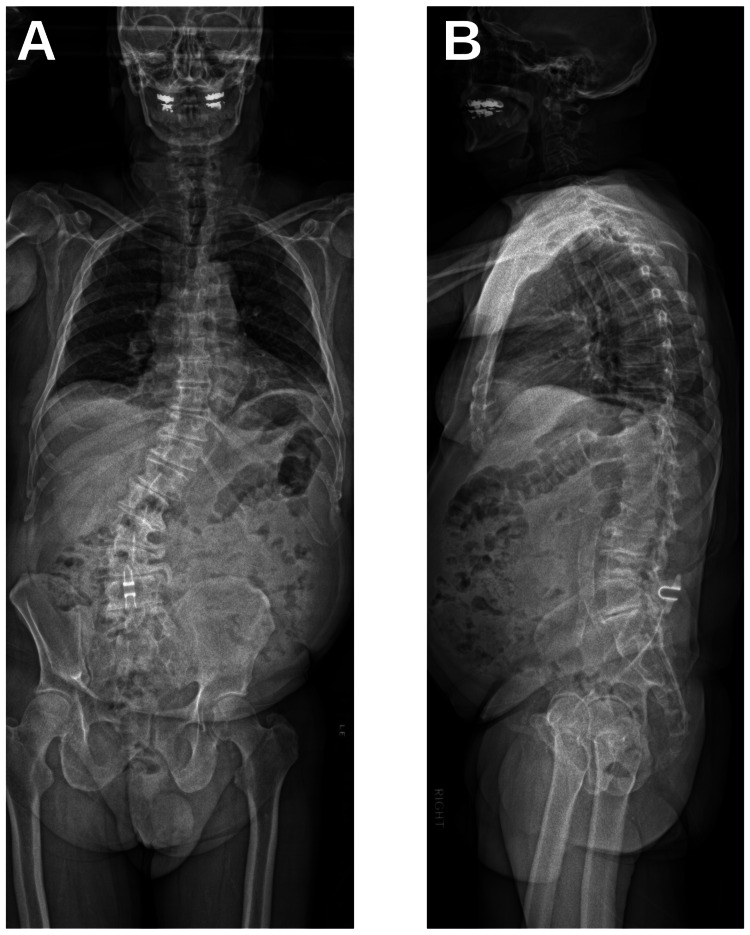
Pre-Operative Radiography Pre-operative coronal (A) and sagittal (B) radiography showing significant scoliotic deformity.

An 82-year-old male presented after undergoing L4-5 decompression and interspinous Co-Flex device implant (Surgalign, Deerfield, IL) with intractable back and leg pain secondary to severe thoracolumbar scoliotic deformity (Lenke 5BN). Pre-operative radiography demonstrated the presence of bridging osteophytes over the left lateral aspect of the L2-5 disc spaces and over the apex of the lumbar curvature with significant neuroforaminal stenosis (Figures 1A, 1B). Relevant pre-operative spinopelvic parameters are listed in Table 1. 

**Table 1:** Pre- and Post-Operative Spinopelvic Parameters

Parameter	Pre-Operative Value	Post-Operative Value (Stage 1)	Post-Operative Value (Stage 2)
L1-5 Cobb angle	38.4°	26.0°	3.4°
Pelvic incidence (PI)	34.0°	39.3°	41.2°
Lumbar lordosis (LL)	19.0°	27.2°	31.4°
PI-LL	15.0°	12.1°	9.8°
Pelvic tilt	15.0°	20.0°	17.0°
C7 plumb line	72.8 mm	30.0 mm	89.0 mm
Head position shift	55.8 mm to left	22.0 mm to left	10.7 mm to the left

Surgical correction

Surgical correction was completed in two stages. Stage one consisted of left-sided lateral osteotomy using an anterior-to-psoas approach (ATP) in the right lateral decubitus position. Significant bridging osteophytes between L2-5 disc spaces were removed using an osteotome and drill, guided by anatomic landmarks and fluoroscopy. Complete diskectomy and interbody fusion were performed at L2-L5 with titanium cages. Stage two, completed the next day, consisted of standard multilevel Ponte osteotomy and instrumented fusion from T10 - pelvis for posterior release of scoliotic deformity and correction of coronal alignment [13]. From T12 to S1, the spinous processes, the lamina, as well as the inferior and superior facet complexes and ligamentum flavum were resected to enable the maximal potential for deformity correction. In addition, posterior lumbar interbody fusion (PLIF) at L5-S1 was performed to provide additional correction and a robust basis for fusion at the bottom of the construct. Relevant spinopelvic parameters after each surgical stage are listed in Table 1.

PROMs and objective outcomes assessment

PROMs (ODI and PROMIS-pain interference) were administered before surgery and at a six-month follow-up. The patient’s mobility data were obtained through remotely enrolling him into a protocol approved by our Institutional Review Board, Pennsylvania Hospital (#843229). The patient was instructed to download "QS Access" (Quantified Self Labs, San Francisco, CA), a free application that exports Apple Health (Apple Inc., Cupertino, CA) data such as steps-per-day. 

A time series of steps taken per day was established with respect to the patient’s date of surgery. The analysis was constrained to a four-year window starting two years before surgery. The time series was z-score normalized with respect to average daily steps over the first year in this window, representing a period where the patient’s daily activity is stable. The normalized time series was smoothed using a seven-day sliding window. An algorithm then automatically classified this time series into distinct epochs representing pre-operative baseline and post-operative recovery by identifying below-average or above-average activity that is sustained for >10 days and reaches certain threshold levels This analysis yields insight into the patient’s pre-operative state of mobility, as well as the timeline of their recovery from surgery.

Surgery outcome

**Figure 2:**
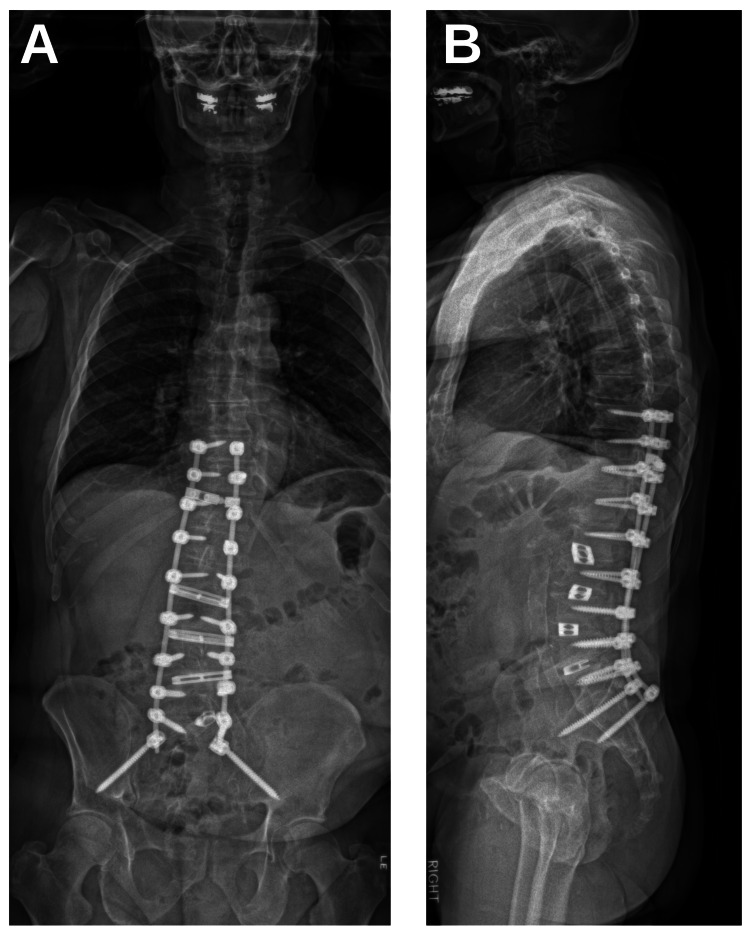
Post-Operative Radiography Post-operative coronal (A) and sagittal (B) radiography shows instrumentation and significant correction of scoliotic deformity.

Procedure time was 5 h and 1 min for stage 1, and 8 h and 14 min for stage 2. Total estimated blood loss (EBL) for stage 1 was 200 mL, and for stage 2 was 3675 mL; the patient remained hemodynamically stable throughout surgery, with transfusion of four units of blood in total. Post-operatively, the patient was monitored in the ICU for several days and was transferred to the neurosurgical floor. On post-operative day 11, the patient had developed wound dehiscence and was taken back to the operating room for wound revision. The patient had no notable complications post-operatively and was eventually discharged 15 days after surgery to an acute rehabilitation facility. Post-operative radiography showed correction to scoliotic deformity and sagittal misalignment (Figures 2A, 2B). 

At six-month follow-up, the patient reported significant improvement in pain symptoms. This was corroborated by PROMs (Table 2). ODI score decreased from 52 to 18 (65% decrease), representing a meaningful decrease in disability as the minimal clinically important difference (MCID) of the ODI instrument in ASD is estimated to be -11% [14]. PROMIS-pain interference score decreased from 67 to 39 (42% decrease), representing a meaningful decrease in pain as the MCID of the PROMIS-pain instrument in ASD is estimated to be -5 points [15].

**Table 2:** Pre- and Post-Operative Patient-Reported Outcome Measures A decrease in ODI, PROMIS-pain, and PROMIS-physical function instruments is understood to be positive, as this denotes a lower disability burden, decreased pain, and increased physical function, respectively. Post-operative PROMs were obtained at six-month follow-up. ODI = Oswestry disability index; PROMIS = patient-reported outcome measurement information system

Parameter	Pre-Operative Value	Post-Operative Value	Percent Change
ODI	52	18	-65%
PROMIS-Pain	67	39	-42%
PROMIS-Physical Function	67	32	-52%

**Figure 3:**
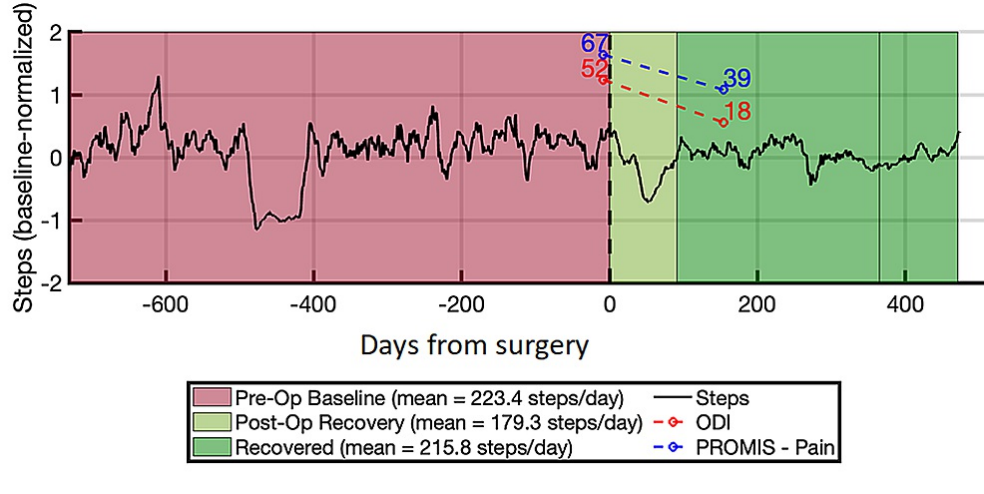
Smartphone-Based Objective Activity Tracking Time series of steps-per-day as obtained from smartphone-based outcomes assessment. Our algorithm classified the patient’s activity history into three distinct epochs: pre-operative baseline, post-operative recovery, and fully recovered state. Overlaid are PROMs scores, which show decrease in pain and disability, but do not reflect the length of post-operative recovery or degree of improvement in patient mobility. ODI = Oswestry disability index; PROMIS = patient-reported outcome measurement information system

Our objective outcome measure successfully used daily steps data obtained from the patient’s smartphone shows to assess the patient’s post-operative recovery course (Figure 3). The patient’s pre-operative baseline was 223 steps/day. The patient’s activity during immediate post-operative recovery dropped to 179 steps/day, which is expected given the extensive nature of ASD corrective surgery. This recovery period lasted for 91 days, after which the patient’s daily steps returned to approximately baseline levels of 216 steps/day. The patient’s average steps/day continues an upward trend, suggesting that the patient’s overall activity level is continuing to increase as a result of their improved mobility after surgery. 

## Discussion

Anatomic considerations

Patients with lateral osteophytes grown over the disk space, causing severe degenerative coronal deformity, can be severely disabling. There are many different surgical options to correct such deformity. One such method is the anterior-to-psoas (ATP) lateral approach, which consists of a lateral approach to the lumbar spine to perform osteotomies and disconnect bridging osteophytes over the apex of the lumbar curvature, then to perform interbody fusion to achieve the restoration of normal alignment in the coronal and sagittal plane. A group from Japan recently published a case report utilizing navigation guidance to perform lateral osteotomy followed by an oblique lateral interbody fusion (OLIF) [16]. In this case report, we achieved osteotomy on the lateral surface of the disk space over the apex of lumbar curvature using solely anatomic landmarks and C-arm fluoroscopy (Siemens, Munich, Germany).

The ATP lateral approach for accessing the lumbar spine is a powerful technique that avoids dissection through the psoas muscle fibers and thereby decreases the force of retraction on the lumbar plexus posteriorly. First, the anterior and posterior vertebral line is marked on the skin based on lateral fluoroscopy. Unlike the trans-psoas approach, in which the skin incision is centered at the center of the disc space of interest, the skin incision for the ATP approach is positioned 2-3 cm anterior to the anterior vertebral line. After dissecting through three abdominal muscle layer, the retroperitoneal space is entered, and the psoas muscle is dissected off from the anterolateral aspect of the vertebral body and retracted posteriorly using a Kitner and a handheld Deaver blade. In this approach, it is critical for surgeons to understand the complex relationship between important anatomic landmarks to avoid catastrophic vascular complications. In the right lateral decubitus position, the position of the great vessels (aorta and inferior vena cava, and their respective bifurcations) is anterior to the vertebral body. Gentle palpation should be performed, and the vessels’ position should be confirmed with the surgeon’s fingers. A retractor should be placed to protect the great vessels from injury. When retracting on the vessels, it is paramount to keep the loose areolar connective tissue (adventitia) attached to the vessels, as it provides a layer of protection. In addition, the ureter, a thick tubular structure that runs cranial to caudal on top of the psoas muscle, should be identified with a visual inspection. Since the ureter is lined with smooth muscle, when gently touched with a handheld suction it will undergo peristalsis. In the upper lumbar spine, other retroperitoneal organs such as the kidney, diaphragm, descending colon become relevant, and these structures need to be visually identified and protected as well. 

Technical nuances

Once the retractors are in place, a sharp quarter-inch osteotome should be used to remove the bridging osteophyte that covers the disk space on the lateral aspect of the lumbar disc. Care should be taken to avoid advancing the osteotome too far, as this can split the vertebral body. An osteotome should be placed above and below the osteophyte. Using fluoroscopy or other navigated technique, the osteotome should be malleted to remove the osteophyte alone. Bridging osteophytes are degenerated cortical bone and, therefore, appear pearly white, as compared to the cancellous bone which is reddish. Once the osteophyte is loosened using an osteotome, either a hand-held rongeur or a large pituitary is used to remove the osteophyte. Once osteophytes are removed, the disc space should be visible. At this point, either a Cobb or a dilator is used to enter the disc space and driven to the opposite side over the lateral annulus using a mallet. Once a complete discectomy is performed, an interbody implant can be inserted laterally into the disc space. For additional levels, the retractor can be repositioned, and osteophyte removal and lateral interbody fusion can be performed using the above steps. 

Objective outcome measures

ASD corrective surgery is a complex procedure and is a significant, life-altering event for patients. When questioned about their expectations of corrective surgery, a majority of ASD patients expected not only an improvement in pain symptoms but also an increased ability to move and exercise [17]. While current PROMs assess post-operative changes in pain and disability, surgeons are currently unable to obtain a true understanding of a patient’s functional improvement in mobility after surgery, and thus unable to determine if a patient’s treatment goals are met.

PROMs, such as the ODI and EQ-5D, are currently only administered in-clinic at the time of post-operative follow-ups. These subjective surveys are not ideal to truly capture the state of a patient’s health and functioning before and after surgery, as imperfect recall and recency bias may skew survey results based on how a patient is feeling on the day of survey administration. These discretized and infrequent data points, operating on the scale of weeks to months, prevent surgeons from obtaining a true picture of a patient's daily functioning. Additionally, the time and human capital needed to administer PROMs results in a significant financial burden, reaching upwards of $150,000 per year. For this reason, 32% of spine surgeons admit to not using PROMs routinely in their clinical practice [18].

In response to these shortcomings, there is a growing body of literature utilizing objective outcome measures, such as assessments of patient mobility, to evaluate spine surgery outcomes. Here, we use activity data (steps-per-day) collected from a patient’s smartphone to gauge their overall mobility and activity levels. Because data points are continuously passively collected, smartphone-based activity monitoring provides a high temporal resolution window into the patient’s pre-operative health and post-operative recovery status. This method of outcomes assessment is also objective and does not depend on subjective patient reporting, which can otherwise bias the data. 

Impact of objective outcome measure in adult spinal deformity

Our results show that while PROMs were an adequate assessment of surgical outcome, smartphone-based activity monitoring yielded more insight into the patient’s post-operative recovery course and how the patient’s functioning and mobility were affected by the surgery, such as the length of the patient’s recovery and the relative improvement in mobility from surgery. The patient’s steps-per-day clearly decreases immediately after surgery and remains low throughout the immediate recovery period (91 days). This post-operative decrease correlates with the expected decrease in mobility while the patient is recovering from surgical intervention. After this recovery period, the patient’s activity levels returned to baseline. Although the patient’s overall activity level did not increase relative to their pre-operative baseline, return to normal mobility with improvement in pain symptoms still indicates successful surgical intervention.

In the future, smartphone-derived mobility data can allow surgeons to precisely track a patient’s recovery in real-time. This is advantageous for all spine surgeries, but especially in ASD corrective surgery, where the extensive nature of surgical intervention requires close monitoring and follow-up. While PROMs alone can measure pain and disability, only an objective measure - such as mobility data - can truly ascertain how a patient’s mobility and functioning is improving, a primary goal in ASD correction. Additionally, inter-instrument comparability between existing PROMs is low, hindering their practical use, while objective measures are, by definition, "ground truth" measurements and thus comparable between patients and between disease states [19].

Limitations and future directions

Our objective outcome analysis methodology, originally developed based on a cohort of patients undergoing decompressive laminectomies, was altered slightly to account for the distinct clinical features of ASD. While this adjustment seems to be successful in measuring post-operative outcomes for a different disease state, more patient data are needed to further refine and validate our work. 

## Conclusions

Lateral osteotomy through an anterior-to-psoas lateral approach can be a powerful technique to achieve the restoration of normal alignment in both coronal and sagittal planes and can be successfully performed using anatomic landmark and fluoroscopy for guidance. Performing an initial lateral approach and subsequent posterior stabilization, as opposed to an entirely posterior approach, can reduce operative time and physical burden on the patient. During the ATP approach, care must be taken to protect the great vessels and ureter, which run anterior to the vertebral body and psoas muscle respectively. Additionally, smartphone-based mobility data are an effective modality to assess pre-operative activity level and post-operative recovery, and allows for remote patient monitoring beyond the routine follow-up schedule. Our findings highlight the potential utility of such data as a primary input in a novel quantitative, longitudinal surgical outcome measure.
